# EDOVE: Energy and Depth Variance-Based Opportunistic Void Avoidance Scheme for Underwater Acoustic Sensor Networks

**DOI:** 10.3390/s17102212

**Published:** 2017-09-26

**Authors:** Safdar Hussain Bouk, Syed Hassan Ahmed, Kyung-Joon Park, Yongsoon Eun

**Affiliations:** 1 Department of Information and Communication Engineering, DGIST, Daegu 42988, Korea; kjp@dgist.ac.kr (K.-J.P.); yeun@dgist.ac.kr (Y.E.); 2 School of Computer Science and Engineering, Kyungpook National University, Daegu 41566, Korea; s.h.ahmed@ieee.org

**Keywords:** void avoidance, depth variance, normalized residual energy, energy balancing

## Abstract

Underwater Acoustic Sensor Network (UASN) comes with intrinsic constraints because it is deployed in the aquatic environment and uses the acoustic signals to communicate. The examples of those constraints are long propagation delay, very limited bandwidth, high energy cost for transmission, very high signal attenuation, costly deployment and battery replacement, and so forth. Therefore, the routing schemes for UASN must take into account those characteristics to achieve energy fairness, avoid energy holes, and improve the network lifetime. The depth based forwarding schemes in literature use node’s depth information to forward data towards the sink. They minimize the data packet duplication by employing the holding time strategy. However, to avoid void holes in the network, they use two hop node proximity information. In this paper, we propose the Energy and Depth variance-based Opportunistic Void avoidance (EDOVE) scheme to gain energy balancing and void avoidance in the network. EDOVE considers not only the depth parameter, but also the normalized residual energy of the one-hop nodes and the normalized depth variance of the second hop neighbors. Hence, it avoids the void regions as well as balances the network energy and increases the network lifetime. The simulation results show that the EDOVE gains more than 15% packet delivery ratio, propagates 50% less copies of data packet, consumes less energy, and has more lifetime than the state of the art forwarding schemes.

## 1. Introduction

Underwater acoustic sensor networks (UASNs) have gained a lot of attention from the research and industrial community due to their vast application areas. The applications of USANs range from marine ecosystem monitoring, seismic activity, offshore exploration monitoring and management, water pollution, to coastal area surveillance. All of those applications require continuous sensing of the aquatic parameters and relaying these sensed parameters towards the onshore station. Sensing and especially communication in the aquatic environment is a quite challenging compared to the terrestrial sensing networks and could not be realized without UASNs.

**Figure 1:**
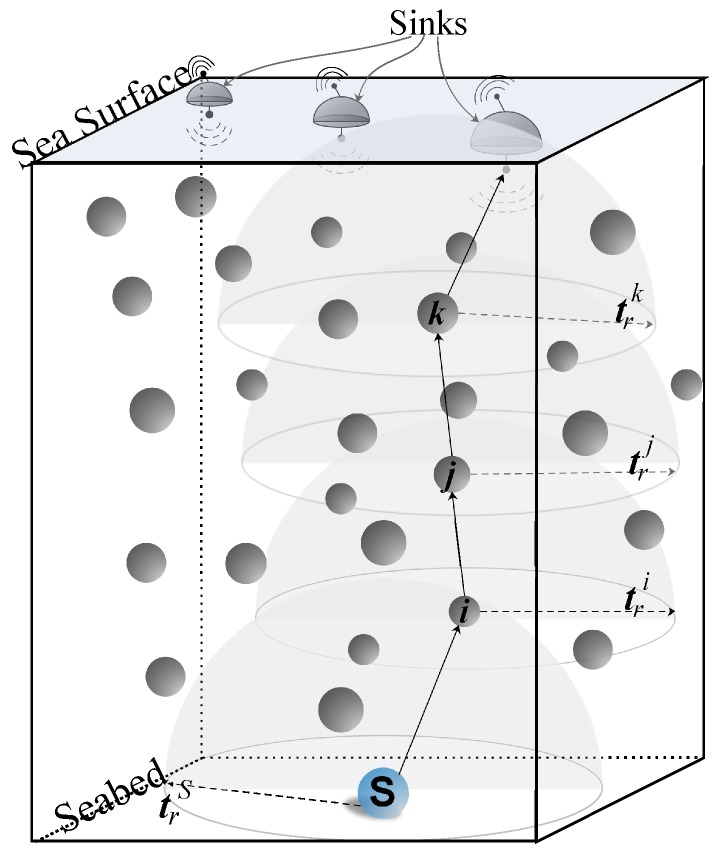
UAN Scenario.

Main elements of the UASNs are the sensing nodes and the sink nodes. The sensing nodes or simply called sensors have the sensing and communication capabilities, they operate on the battery power, and usually, they rest at the bottom of the sea and/or anchored to the seabed. Normally, the anchored nodes in UASNs are the nodes that know their location information [1]. However, here the concept of anchored node is the node that is attached with the cord, which is connected to the anchor. In addition to the sensing and communication, these sensor nodes can store small size contents due to the limited storage capacity of the node. Sensor nodes in the aquatic environment can communicate using the acoustic [2,3], electromagnetic/radio [4,5], or optical communication modes [6,7]. Sensor nodes commonly use the acoustic communication mode. However, the acoustic communication mode inherits some peculiar characteristics. Acoustic communication has a very long communication range, a very long propagation delay, high absorption and path loss, high energy requirement for the transmission and reception of the acoustic signal, limited bandwidth due to high error rate, and so forth [8,9]. On the other hand, the sink nodes or simply Sinks float over the sea surface. Sinks use multiple interfaces, one interface to communicate with the underwater deployed nodes (e.g., acoustic communication mode) and the other interface to connect with the onshore monitoring and control center (e.g., radio communication mode). The sensor nodes forward or route the sensed data towards the Sink which further relays this data to the onshore monitoring stations. The generic UASN scenario is shown in Figure 1, where multiple sinks are floating over the sea surface. The underwater acoustic sensors are deployed under water with the acoustic transmission range of $t_{r}$. In that scenario, node *S* with transmission range $t_{r}^{S}$ communicates the sensed data towards the sink through node the nodes *i*, *j*, and *k* with transmission ranges $t_{r}^{i}$, $t_{r}^{j}$, and $t_{r}^{k}$, respectively.

As stated earlier, the electromagnetic or radio and optical communications modes can also be used in the underwater environment; however, they are not suitable due to different aquatic characteristics. Electromagnetic signals at radio frequencies are preferable for the terrestrial communication because they consume low energy, offer high bandwidth, small propagation delay, and fairly low signal attenuation. Hence, the Sinks use the radio communication mode to connect the onshore stations. However, radio communication in the aquatic environment is not suitable because of the high attenuation that results in the severe absorption because of the water conductivity. In result, the radio communication has a limited small transmission range in the aquatic environment. Similarly, the optical communication requires a very precise line of sight between *receiver* and *Sender* node pair as well as the clear visibility. These two constraints are difficult to meet because of the water current and its turbidity. Hence, the optical communication fails to achieve the high data rate communication over the long range. Because of these challenges and limitations, the acoustic communication mode is widely used in the underwater networks. However, the data collection and dissemination solutions for the terrestrial networks can not directly be applied in the underwater network due to due to its inherent limitations.

There are many routing protocols in the literature that tackle and consider the intrinsic acoustic parameters (e.g., high energy consumption, long propagation delay, large error rate, low bandwidth and so forth) to perform routing decisions. There are many ways the authors have classified the routing protocols in the literature, however, here we classify those protocols into two main categories, Location or localization-base and depth based routing protocols. In Location or localization based routing protocols, each node knows its own three-dimensional location, the location of the destination node(s) or sink(s), and sometimes the location of the neighboring nodes [10,11,12,13,14]. Each packet contains the location of the destination node as well. The main objective of these routing protocols is to directionally forward the data packet towards the sink(s) to minimize the propagation delay, reduce energy consumption and utilize minimum bandwidth by restricting the broadcast of multiple copies of the data packet. Another category of routing protocols consists of the routing schemes that do not use any location information but the depth of the node [15,16,17,18,19,20]. Node’s depth information can be estimated locally by the node itself from the pressure sensor, and it does not require any additional control overhead. The packet does not contain any destination information because it is assumed that the packet needs to be forwarded towards the surface. Additionally, the packet contains the depth of the relaying node. In the context of this paper, we focus on the depth based routing schemes.

The depth based routing schemes minimize the propagation delay by distributively selecting the next forwarder node among the neighbors that has the smallest depth than the packet forwarder or *Sender* node. All the other nodes at the depths between the packet forwarder and the lowest depth node will simply discard or suppress the data packet transmission [15]. This packet suppression at the intermediate nodes minimizes the energy consumption and implemented using the holding time. In the holding time strategy, each node computes its holding time when it receives the data packet and the holding time computation simply involves the depth information of the node(s). Before computation of the holding time, each node must check whether its depth is smaller than the depth of the data packet forwarding node. If a node has the smaller depth, then it estimates the holding time. Otherwise, it simply discards the packet. Holding time of the smaller depth node(s) that the node(s) with the smallest depth in the neighborhood of the data packet sender must have the smallest holding time among all neighbors. After computation of the holding time, it initiates the timer. Once the holding timer expires, the node verifies whether it has received additional copies of the same data packet or not. If it did not receive any additional copy, then it further transmits the packet. Otherwise, it suppresses the transmission and removes the copy of the data packet from its buffer. To ensure that the smaller depth neighbor must forward the packet, its holding time must be shorter than rest of the neighbors. Along with the depth information, there are some depth based routing schemes that consider not only depth but other metrics to forward the data packets or compute the holding time. These metrics not only involves the depth but also the energy of the node, link quality, depth information of the next hop forwarders and so forth [16,17,18,19,20]. The propagation delay in the depth based routing scheme can be minimized if the node with lesser depth forwards the data packet because that node is closer to Sink. However, this depth based forwarding process may penalize the less depth nodes, and all the communication is forwarded through the same set of nodes. In result, the battery power of these nodes will drain very quickly and create the void region. The phenomenon of the void region is simply described as the area in the network where there is no node available to further the communication towards the destination node that is Sink. Hence, any node at the edge of the void region fails to forward the data packets. In order to avoid the communication through edge node of the void region, the schemes in literature consider the depth differences between the sender, receiver and one-hop neighbors of *receiver* nodes. It assures that the potential forwarder must have the next hop node, not the void region. However, this type of schemes do not avoid the formation of the void region and do not provide any energy balancing in the network. Therefore, the routing schemes should avoid communication through void region and balance the energy by distributing the data broadcast load evenly on different nodes in the network. The lowest depth nodes with small residual energy must not be penalized. In this manner, we can delay the creation of the void region. However, if there is already a void region in the network, then the routing scheme must also divert the communication around the void area.

By keeping all those features under consideration, we have proposed an Energy and Depth variance-based Opportunistic Void avoidance (EDOVE) scheme in this paper. EDOVE uses the depth difference information between the data packet receiving node (simply we call it *receiver* node in this context) and the sender data , forwarding or relaying node (call it *Sender* node in the context of this paper.) as well as the neighbors of the *receiver* node. EDOVE prioritize the receiver node to become the potential forwarding candidate, if:

- it has maximum residual energy among its one hop neighbors.
- it has one or more than one neighbors with less depth.
- it has more number of neighbors with depth less than the *receiver* node and their normalized depth variance should be larger.

The above mentioned characteristics select a potential forwarder that has multiple nodes with lower depth to avoid data packet collision at the next hop, does not penalize the low battery power nodes, and avoids the potential void region.

The rest of the manuscript is organized in the following manner: The literature review that is related to the proposed EDOVE is summarized in Section 2. In Section 3, holding time and working principle of EDOVE is described. Simulation analysis of EDOVE is compared to the state of the art scheme in terms of end-to-end delay, energy consumptions, the number of copies of data packets, and hop count, packet delivery ratio, dead nodes, etc., are discussed in Section 4. Finally, the overall conclusion extracted from the discussion is presented in Section 5.

## 2. Previous Works

There have been many routing schemes proposed to achieve efficient routing in the underwater acoustic networks[21,22,23]. In literature, there are many schemes that avoid the duplicate packet forwarding using the backoff timers in the flooding-based underwater acoustic networks, as in [24]. When a node receives the data packet, it initiates the backoff timer and holds the communication of that packet. If it receives a certain number of copies of the same data packet, which is floor(2.5), the node simply discards the packet. The timer is selected randomly between the predefined minimum and maximum backoff duration that is 5 s and 65 s. This scheme does not use any additional network parameter in the forwarding decision. The above work was extended by authors in [25], where they used hop count information, additional to the backoff, to better decide which packet should be considered duplicate and discarded. There are many similar works in the literature that involve the backoff timers, however, in this section, we discuss the depth based opportunistic void avoidance schemes, which are closely related to our proposed EDOVE scheme.

As discussed in the previous text, the depth information is the prime metric that is used by the routing protocols. It is estimated locally from the reading of the pressure sensor. Data packets are forwarded from the higher depth nodes to the lower depth towards the surface where sink(s) that are floating on the water surface. In addition to the depth information, several other metrics have been adapted by the routing schemes to efficiently forward the data packets to sink(s). Depth based routing schemes are further categorized into two categories; *sender initiated* (The next hop forwarder of the data packet is selected by the *Sender* node) [17,19] and the *receiver initiated* (where the data packet receiver decides whether it forwards the packet or not) [15,16,18,20].

In the *sender initiated* protocols, the data packet *Sender* node selects the next hop relay node, and the information of the next forwarder is added in the data packet. Once the node receives that data packet, it easily determines from the header information of the data packet that node has to further relay the packet or not. On the other hand in the *receiver initiated* routing protocols, the data packet receiving nodes distributively contend to be the next forwarder. These nodes compute their respective holding times, and the potential forwarding candidate(s) has/have very small holding time compared to the other nodes. The node with smallest holding time forwards the data packet, and when the other nodes receive a copy of the same data packet while their holding time is still on, the simply discard the transmission [15]. In this manner, the *receiver initiated* forwarding schemes suppress the data packet broadcast. Hence, the data packet broadcast suppression depends upon the variance of the holding times computed by the nodes. In this section, we merely focus on the *receiver initiated* depth based routing schemes because the proposed EDOVE belongs to that routing category.

**Figure 2:**
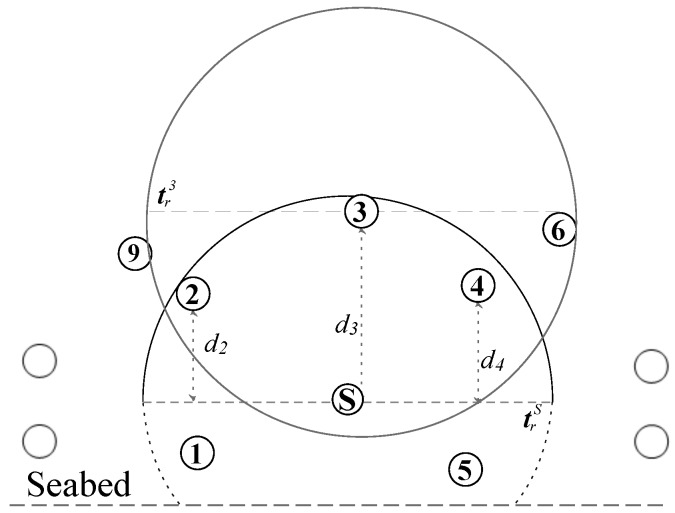
Depth based routing scenario.

In [15], authors proposed a holding time based packet suppression scheme where packet receiving nodes take the forwarding decision using their depth information, called Depth Based Routing (DBR). All the nodes that are in transmission range of the sender and have smaller depths are candidate forwarders. Rest of the nodes that receive data packet and are placed at the larger depth will simply discard the data packet. Consider the DBR scenario in Figure 2, where *Sender* node *S* either sends or forwards the data packet. All nodes within the transmission range of *S*, $t_{r}^{S}$, e.g., nodes 1 to 5, receive the data message. The receiving nodes 2, 3 and 4 with depths $d_{2}$, $d_{3}$ and $d_{4}$, respectively, are the potential forwarding candidates because their depths are less than the depth of *S*. According to DBR, node 3 is a preferable forwarder due to the lowest depth among all the other nodes. Conversely, the nodes 1 and 5 have higher depth than *S*. Hence, they will discard the packet. All potential forwarders in DBR compute their holding time and the smallest depth node has the very short holding time. DBR computes holding time using the depth difference between *Sender* and *receiver* nodes. Hence, the nodes with smaller depth and very large depth difference will always have the short holding time, such as node 3 in the scenario shown in Figure 2. In other words, among all the candidate nodes, the node with minimum depth always forward the packet in the upstream direction. Whenever the same set of senders forward the data packet(s), that particular set of node(s) in the neighborhood with minimum depth are penalized and die out earlier. The number of redundant packet transmissions increases with the increase in the network density because the delay difference between their holding times become negligible. Accordingly, excessive energy is consumed, and more packets fail to reach the sink node.

Authors in [16], proposed an Energy Efficient Depth Based Routing (EEDBR). EEDBR computes the holding time using node’s residual energy and depth information to forward the packet. Every node maintains the neighborhood list where it records the node identifiers (IDs) along with their residual energy. In EEDBR, the data sending node selects the set of potential forwarders with smaller depths from the neighborhood list and IDs of those potential forwarders are added in the data packet. In addition to that, the required packet delivery ratio set by the application is also added in the data packet header. When a node receives the data packet, it first checks whether it is in the potential forwarder set or not. If a node is not in the potential forwarder set, then it simply discards the packet. On the other hand, if a node is in the potential forwarder set, it compares its residual energy with the residual energy of its neighbors in the neighborhood list. In case, the node has maximum residual energy among its neighbors; it will forward the packet immediately. Rest of the potential nodes with less residual energy, compute and start their holding timer and defer the transmission. If any node during its active holding timer receives a copy of the same data packet, it will compute a random number. This random number is used to decide whether to forward the data packet or not once the holding timer expires. If the random number is less than the packet delivery ratio specified in the data packet and the node received multiple copies of the data packet, then it simply discards the packet. A node only forwards the data packet, after its holding timer expires, if any of the following conditions meet; either its computed random number is larger than the delivery ratio specified in the data packet header, or it did not receive any other copy of the same data packet. EEDBR fails to suppress multiple transmission of the packets and the number of packet transmissions can greatly increase in the dense network scenario.

Another depth, hop count, and link quality based routing scheme, called location-free link state routing (LLSR), has been proposed in [17]. The proximity of a node to the Sink is deduced from the hop-count metric and authors assume that this metric avoids the communication through the void region. The beacon dissemination phase started by the Sink with *zero* hop count value and any node that receives this beacon message just increments and records the received hop count value and then further disseminates the updated hop count in the same beacon message. In addition to the hop count metric, a node also stores the path quality towards the sink. Subsequently, each node periodically broadcasts its depth, path quality, and hop-count metrics during the beacon dissemination phase. Each node maintains the 1-hop neighbor table to make the forwarding decision. The data packet forwarding node first checks the lowest hop-count node neighbor to select it as a potential forwarder. In a tie situation, where two or more than two neighbors have the minimum lowest count, the decision is taken based on the path quality. If still that tie persists, then the next tie breaker metric is the depth of the node. The lowest depth node will get the chance to forward the data packet. The packets will be forwarded through the same set of nodes that have lowest hops, good path quality, and smaller depth. In result, it will penalize the low energy nodes and they will die out quickly. Moreover, the beaconing overhead to maintain the soft-state information is very high and that drastically consumes the network energy.

Another *receiver initiated* void avoidance routing protocol, called inherently void avoidance routing (IVAR), has been proposed in [18]. IVAR forwards packets towards Sinks using two routing metrics: depth and hop count of a node. In order to maintain the hop count at each node, all Sinks periodically broadcast the beacon message. This beacon message is forwarded as in [17]. Each data packet includes the hop count information. When a data packet is forwarded by the node, then all the receiving nodes that have the hop count smaller than the one in the data packet are eligible candidates. These forwarding nodes compute their holding time using their respective depth information. In IVAR, the nodes at the edge of a void region simply discard the transmission, because their hop count is either equal or greater than the one in the received data packet. IVAR avoids the void region and the communication is carried out through the nodes with the small hop count only and it fails to achieve and balance the energy efficiency.

The authors in [19] improved the shortcomings of the IVAR and proposed a *sender initiated* opportunistic void avoidance routing (OVAR). These shortcomings include; the duplicate packet transmission that is resulted by the small difference in the holding times of the candidate forwarder and the hidden terminal problem of IVAR. OVAR adapts the same periodic beaconing method to maintain information of the 1-hop neighbors. This 1-hop adjacency information at each forwarder helps it to select the next candidate forwarders. The candidate nodes are selected in a proximity to avoid the hidden node problem and reduce duplicate packet transmission. In the above-discussed forwarding protocols that use beacons to update proximity graph, e.g., LLSR, OVAR and IVAR, neighborhood information freshness depends on the beacon interval. The lower the beacon interval, the higher the neighborhood information accuracy and better the forwarding decision. However, the low beacon interval has severe impact on the battery power. Recently, a *receiver initiated* depth based routing protocol, named weighting depth and forwarding area division DBR (WDFAD-DBR), has been proposed that avoids the void hole problem in UWSNs [20]. It achieves energy efficiency in the dense network scenario by suppressing the duplicate data packets and reducing end-to-end delay by considering the two-hop distance value in the holding time calculation. Routing in WDFAD-DBR is performed into two phases: First, the forwarding region is divided into one primary and two auxiliary forwarding areas to increase the chances of packet delivery. Second, it computes the weighted sum of the depth differences of two hops to minimize the packet duplication. This two hop information prevents the void hole encounter, however, merely two hop depth difference does not eliminate the chance of void hole encounter. The probability of packet drop and end-to-end delay increases with the increase in the network density because the holding time does not use any node density information.

**Figure 3:**
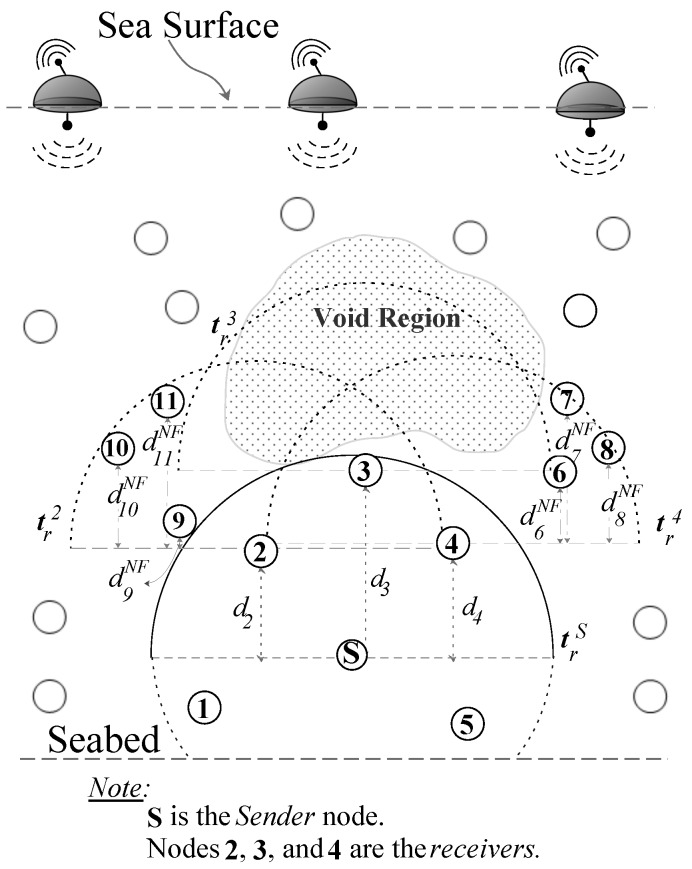
WDFAD-DBR routing scenario.

Figure 3 shows the void region scenario where node *S* sends the data packet and nodes 1–5 are within transmission range of node *S*, $t_{r}^{S}$. Nodes 2–4 are the forwarding candidates and the nodes 1 and 5 simply drop the packet because they have larger depth than *S*. As node 3 has no next hop forwarder, hence, it does not compute the holding time and simply discards the packet. However, nodes 2 and 4 compute their holding times using two hop depth difference; depth difference to *S* and maximum depth difference to their neighbors with the lower depths, ($d_{2}$ and $d_{11}^{NF}$), and ($d_{4}$ and $d_{7}^{NF}$), respectively. Let, node 4 has the largest two hop depth difference, which makes it an appropriate forwarder. It is worth noting that the neighbors of node 4 are in a close proximity. Therefore, once the node 4 relays the packet and its neighbors contend to further forward the packet, it will increase the probability of duplicate packets, collision, and large energy consumption. Similarly, if the residual energy of node 4 is very small, then every time *S* forwards the data packet through node 4 and it consumes the energy of node 4 and its neighbors and eventually will widen the void hole immediately.

### Motivation and Contributions

Most of the depth based forwarding schemes in the literature just consider depth plus energy of the node itself to forward the packet. There are couple of schemes that consider link quality and hop count. However, they need to exchange a lot of beacons or short messages to get the current link quality and to update the hop count in the link breakage situation. This beacon control overhead has a drastic impact on a node’s battery and the overall network lifetime.

Inspired from the above discussion, we proposed a novel EDOVE for UASNs. EDOVE computes the holding time of candidate forwarders by keeping the following points under consideration.

1. Avoid using constant weighting factor(s) to compute the holding time because the estimation of the optimal value quite difficult in the unpredictable and error prone topology network, e.g., α weighting factor was used by WDFAD-DBR.
2. Select the forwarder from the 1-hop neighbors of the data *Sender* node (also known as the *data receiver nodes*) that has smaller depth relative to the sender node and has more residual energy. To achieve this, we use depth difference between sender and its 1-hop neighbors (the *first hop depth difference parameter*) and it is prioritized using the normalized residual energy of the potential forwarder. The main objective of prioritizing the smaller depth potential forwarder with larger residual energy is to achieve energy efficiency, energy balancing, and disseminating data further in the network. In result, it extends the network lifetime and minimizes the data broadcast overhead.
3. Next, choose the forwarder that has larger depth difference relative to its neighbors, which is the *second hop depth difference,* and has more depth variance among its neighbors. The second hop depth parameter is scaled with the depth proximity variance of the neighbors of the potential forwarder node. Any potential forwarder that has the large depth proximity variance becomes the potential forwarder and it intends to avoid the packet collision and prevents the void region when it further forwards the packet.
4. The adaptive weighting factors, based on 2 the residual energy and 3 the depth variance, with one and two hop depth difference parameters, significantly scale the holding time of the receivers to avoid multiple data packet transmissions.

In the next section, we briefly discuss our proposed EDOVE scheme and next we discuss the simulation results, which shows that EDOVE improves energy efficiency, increases packet delivery ratio, and network lifetime.

## 3. Proposed EDOVE Scheme

In this section, we present the detailed discussion of our proposed EDOVE scheme. EDOVE avoids the void region by distributively selecting the forwarder with large residual energy and having multiple neighbors that are largely distributed depth-wise. The proposed scheme is based on and compared with the WDFAD-DBR, which avoids the void hole and uses the holding time $\left(T_{h}\right)$ to suppress the duplicate packet transmission. Void hole avoidance is achieved through prioritizing the forwarders that have one or more than one neighbors with the smaller depth. WDFAD-DBR uses a constant weighting factor, α∈(0,1), to prioritize either first hop (sender *S* and receiver *i*’s depth difference, $d_{i}=\left(z_{S}-z_{i}\right)$ and second hop depth differences (receiver and its minimum depth next hop neighbor *j*, $d_{i}^{NF}=\left(z_{i}-min\left(z_{j}\right)\right)$, *H*.

$$H=\alpha \left(d_{i}\right)+\left(1-\alpha \right)\left(d_{j}^{NF}\right)$$

In addition to that, a maximum acoustic delay between all the receivers, β, is multiplied with *H* to compute the holding time at receiver *i*, $T_{h_{i}}$:

$$T_{h_{i}}=\beta \left(t_{r}^{S}-H\right)$$

where $\beta =\frac{2t_{r}^{S}/v_{sound}}{\rho }$, $\rho \in (0,t_{r}^{S}]$, $t_{r}^{S}$ is the transmission range of *S*, and $v_{sound}$ is the sound velocity in the aquatic environment.

Opposite to WDFAD-DBR, the proposed EDOVE computes $T_{h_{i}}$ using the weighted two hop depth differences *H*, the normalized residual energy of node *i*, $e_{i}$ and the next hop depth difference variance, $\delta_{i}$. Hence, the resultant $T_{h_{i}}$ prioritizes the receiver that has more available energy, large depth difference to *S* and receiver with large depth difference variance to minimize further data collisions. As a result, energy efficiency is achieved by avoiding the packet collision with void avoidance and increasing the network lifetime.

### 3.1. Problem Statement

Consider the underwater network scenario in Figure 3 with sender *S*, receivers with residual energy $E_{i}$ within the $t_{r}^{S}$ of *S*, and receiver with small hop neighbors. The sender *S* either generates or relays the data packet and all the receivers simply receive that packet and that packet has to be relayed through any of these receivers. However, these receivers are at the edge of the void zone and the forwarding algorithm must avoid selecting the relay that fails to further relay the packet due to the void zone. Furthermore, these receivers have different residual energy and the forwarding strategy has to select the receiver with large residual energy to balance network-wise energy consumption. Selection of one receiver among multiple potential ones is done through the holding time of the receiver *i*, $T_{h_{i}}$.

When receiver *i* receives a data packet, it computes and starts the $T_{h_{i}}$. While $T_{h_{i}}$ is running and *i* receives another copy of the same packet, it suppresses the packet transmission. Nonetheless, *i* forwards the data packet if it does not receive any more copies of the data packet until the $T_{h_{i}}$ expires. However, the computation of $T_{h_{i}}$ must consider the long propagation delay between the receivers and must involve the weighted depth differences, the normalized residual energy of the receiver, and variance of the depth differences of the next hop neighbors of *i*. So, the duplicate packet broadcast along with the void hole can be avoided. However, the impact of this long holding time may result in the long end-to-end delay.

Next, we discuss the proposed EDOVE and its holding time computation on how it suppresses the additional data packet and avoids the void area in the underwater network.

### 3.2. Preliminary Notations

**Figure 4:**
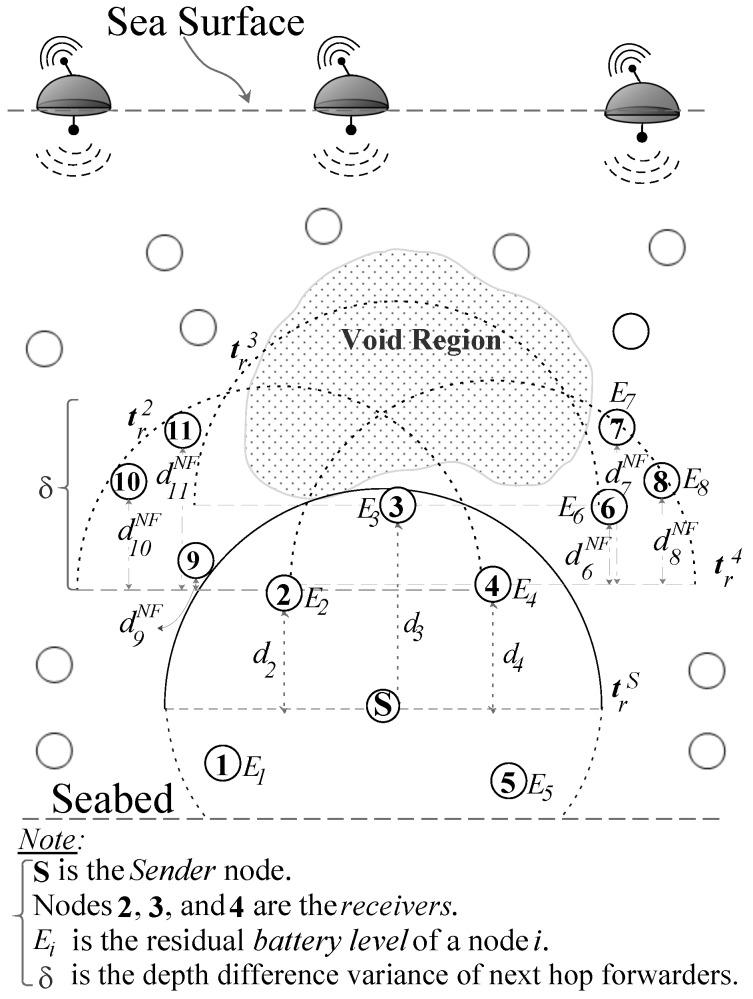
EDOVE network parameters to compute holding time.

EDOVE uses the notations in the holding time computation and the forwarding algorithm, and these notations are briefly described below with reference to the EDOVE network scenario shown in Figure 4.

- *Network size |N|*: is the total number of nodes N with the acoustic communication capabilities that form the network, is termed as the network size.
- *The set of receiver nodes $\kappa_{S}$*: consists the nodes that are within the transmission range of *S* and have depths less than the depth of *S*:
  (1) $$\kappa_{S}=\left\{j\in N|z_{j}<z_{S}\wedge dist_{\left(j,S\right)}\leq t_{r}^{S}\right\}$$
  where, $dist_{j,s}$ is euclidean distance between node *j* and *S*, and $z_{j}$ and $z_{S}$ are the depths of node *j* and *S*, respectively. Figure 4 shows that the nodes 1 to 5 are the neighbors of *S* and $\kappa_{S}=\left\{2,3,4\right\}$.
- *Next hop forwarders of node i, $\kappa_{i}^{NF}$*: are the nodes that lie within node *i*’s transmission range and having depths less than the depth of *i*:
  (2) $$\kappa_{i}^{NF}=\left\{j\in N|z_{j}<z_{i}\wedge dist_{\left(i,j\right)}\leq t_{r}^{i}\right\}$$
  where, $z_{j}$ and $z_{i}$ are the depths of next hop node *j* and *receiver* node *i*, respectively. The next hop forwarder of receiver 4, $\kappa_{4}^{NF}=\left\{3,6,7,8\right\}$, as shown in Figure 4. However, $\kappa_{3}^{NF}=\emptyset$.
- *Residual energy of node i, $E_{i}$*: is the current available battery level of node *i*.
- *Normalized depth variance of node i, $\delta_{i}$*: is the normalized variance of the depth differences of all the nodes in $\kappa_{i}^{NF}$.

The above discussed notations are used by the EDOVE to compute holding time and the forwarding algorithm that are briefly described below.

### 3.3. Estimation of $T_{h_{i}}$

Let, node *S* sends or forwards the data packet and node *i* within its $t_{r}^{S}$ receives that data packet. Then, the node *i* first compares its depth to the depth of *S*. If *i* has the depth larger than that of *S*, $z_{i}>z_{S}$, then it simply discards the data packet. On the other hand, if node *i*’s depth is less than the depth *S* and has one or more than one neighbors with smaller depth (i.e., $z_{i}<z_{S}$ and $|\kappa_{i}^{NF}|\geq 1$), then it computes the holding time $T_{h_{i}}$. However, if node *i* has no neighbor at the depth lower than *i*, then it also discards the data packet. The holding time of node *i* should be very small compared to its neighboring nodes if it has the following characteristics:

- larger residual energy than its neighbors.
- larger depth difference than sender.
- larger depth difference to its minimum depth neighbor.
- many neighbors with large variance in their depth differences.

EDOVE considers all the above mentioned characteristics to calculate the holding time of $i\in \kappa_{S}$ as follows:

(3) $$T_{h_{i}}=\frac{2\times t_{r}^{S}}{v_{Sound}}\left(H\right)$$

where,

(4) $$H=e_{i}\times \left(1-\frac{d_{i}}{t_{r}^{S}}\right)+\left(1-\frac{max(d_{j}^{NF})}{t_{r}^{i}}\right)\times \delta_{i},$$

$$j=1,\ldots ,|\kappa_{i}^{NF}|,$$

$$v_{Sound}\approx 1500\;m/s.$$

Equation (4) consists of two parts. First part prioritizes a node from the 1-hop neighbors of the data sender that has smaller depth with larger depth difference $d_{i}$ relative to the *Sender* node and large normalized residual energy, $e_{i}$, which is explained later in this section. The main objective of prioritizing the smaller depth potential forwarder with larger residual energy is to achieve energy efficiency, energy balancing, and disseminating data further in the network. In result, it extends the network lifetime and minimizes the data broadcast overhead. Next part of Equation (4) considers the depth difference between the potential forwarder *i* relative to its neighbors, $max(d_{j}^{NF})$, and depth variance among its neighbors $\delta_{i}$. Any data receiving node that has the large depth difference to its lowest depth neighbor and has large depth variance becomes the potential forwarder. This part of the expression intends to avoid the packet collision and prevents the void region when it further forwards the packet.

The factor, $e_{i}$, in Equation (4) is the normalized residual energy of *i* computed using the residual energy of all the neighbors of *i*, $\kappa_{i}$.

(5) $$e_{i}=\frac{E_{max}-E_{i}}{E_{max}-E_{min}}$$

$$\begin{array}{cc} \mathrm{where}, & \\ E_{i} & =\mathrm{Residual}\;\mathrm{Energy}\;\mathrm{of}\;\mathrm{node}\;i,\\ E_{max} & \neq E_{min},\\ E_{min} & =min\left(E_{k}|\forall k\in \kappa_{r}\right),\\ E_{max} & =max\left(E_{k}|\forall k\in \kappa_{r}\right),\mathrm{and}\\ e_{i} & \in [0,1]. \end{array}$$

In case, $E_{max}=E_{min}$, then $e_{i}=1.0$. The value of $e_{i}$ will be smaller for the node *i* with large residual energy and that node will be prioritized to forward the data packet. Similarly, the second factor, $\delta_{i}$, in Equation (4) is the normalized variance of the depth differences of the next forwarding nodes of *i*, $\delta_{i}$.

(6) $$\delta_{i}=1-\left(\frac{\frac{1}{|\kappa_{i}^{NF}|-1}\sum_{j=1}^{|\kappa_{i}^{NF}|}{\left(d_{j}^{NF}-\mu \right)}^{2}}{\mu }/t_{r}^{i}\right)$$

where,

$$\mu =\frac{1}{|\kappa_{i}^{NF}|}\sum_{m=1}^{|\kappa_{i}^{NF}|}\left(d_{m}^{NF}\right)$$

The value returned by Equation (6) will be smaller for the receiver *i* that has the large normalized depth difference variance, and that node will be prioritized to act as the forwarder node. Hence, the proposed holding time in EDOVE will prefer the node that has large residual energy and has multiple smaller depth nodes with large depth variance to further avoid the data packet collision.

Since, computation of the holding time requires residual battery power and distance information of its one-hop neighbor, therefore, each node exchanges this information in the neighbor request (NBR_REQ) and neighbor acknowledgment (NBR_ACK) packets, as in [20]. This information is tabulated in the neighbor table (NBR_TABLE). A detailed working principle of our proposed EDOVE scheme is illustrated in Algorithm 1.

It is very obvious from the Algorithm 1 that once a node *i* receives the *data* packet, it first checks whether the *data* packet is already in packet queue, *Q*, or not. If *i* did not already process the *data* packet, then it verifies the timer for that packet. Next, *i* compares its depth to the one in the *data* packet. If *i* is within the receivers of *data* $\kappa_{i}$ and has more number of neighbors with less depth $\kappa_{i}^{NF}$, then it computes the $T_{h_{i}}$. Otherwise, *i* just updates the neighborhood table and drops the *data* packet. When *i* computes the $T_{h_{i}}$, then it sets this holding time value to the timer ($Timer_{data}=T_{h_{i}}$) and starts. While the *data* timer is running and *i* receives another copy of the packet, then it just discards the packet and updates the number of copies received count. Once the $Timer_{data}$ is triggered, *i* checks whether it a single copy or multiple copies of the *data* packet. The node *i* drops the packet if it receives multiple copies. Otherwise, it forwards the *data* packet with the updated depth, energy, etc. parameters in the packet.

**Algorithm 1:** Proposed EDOVE Scheme.
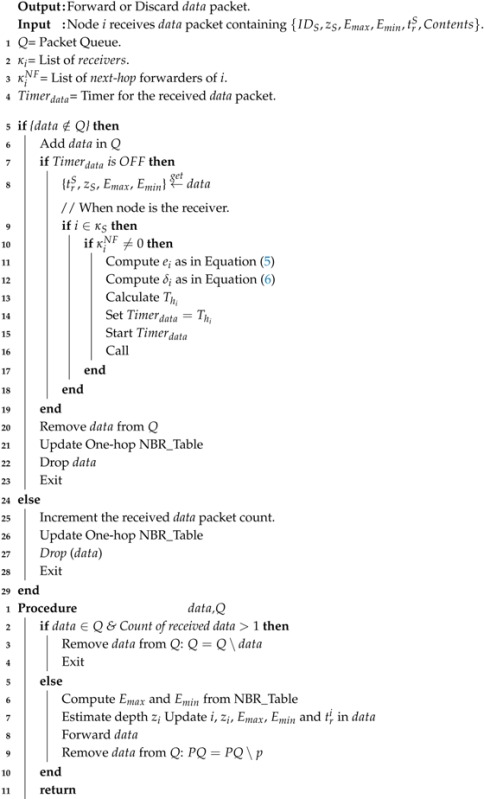


## 4. Simulation Analysis

The detailed performance comparison of the proposed EDOVE scheme in contrast to the conventional WDFAD-DBR scheme through simulations is presented in this section. During simulations, we considered the three-dimensional network area of 1 km length and width and 2 km of depth, (for example, $X_{max}=Y_{max}=1$ km and $Z_{max}=2$ km). A varying size network of 60, 90, …, up to 180 nodes has been considered in the simulations. All nodes are homogeneous in terms of transmission range, which ranges from 600 m to 1000 m. These parameters are sufficient enough to demonstrate the network’s spares and denseness. During all simulations, the data generating node or source node is placed at the bottom of the ocean and the sink(s) are placed at the sea surface. In this scenario, the impact of depth as well as multi-hop forwarding on the depth-based proposed solutions can easily be investigated. Rest of the nodes are randomly deployed between source and sink node(s) in each simulation scenario. These nodes serve as the data forwarders or relaying nodes between data source and the sink(s). This typical scenario explicitly demonstrates, how the data relaying load among forwarders is distributed by the forwarding scheme.

In first simulation trial for 60 nodes, except data source, all nodes are randomly deployed in the network area. In the next trial for 90 nodes, the first 60 nodes’ position is un-altered and only the next 30 nodes are randomly placed in the network. There are five sinks deployed over the surface of the network area. It is assumed that the whole network is static throughout the simulation duration. Each node is assigned initial energy $E_{0}$ that is equal to $E_{min}+rand\left(E_{rand}\right)$, Where $E_{min}=60$ J and $E_{rand}=20$ J. We use the simplified energy consumption model at each node. Energy consumed by a node to transmit *m* bits at distance *k* meters is presented as:

$$E_{t_{x}}\left(m,k\right)=E_{ele}\times m+E_{amp}\times m\times k^{2}$$

where $E_{ele}$ is the energy required to send 1 bit and $E_{amp}$ is the acoustic amplifier energy or acoustic wave attenuation coefficient. Energy consumption at the node, which receives *m* bits is represented as: $E_{r_{x}}\left(m\right)=E_{ele}\times m$. The node’s sending, receiving, and idle energies are 50 W, 158 mW and 58 mW, respectively.

The data, control traffic, and acoustic network parameters used in the simulations are as follows: The header and payload size of data packet is 88 and 576 bits, respectively. Neighbor request and acknowledgment are 48 bits. The common header of 88 bits is used for all packet types in the simulation. Additionally, the acoustic propagation delay of 1500 m in the aquatic environment and the data rate of $16\times 10^{3}$ bits per second have been considered in the simulations. In last, at MAC layer, the pure ALOHA is used because of its is not susceptible to delays and does not use any collision detection and avoidance mechanism.

As stated earlier that the source node is deployed at the seabed and it generates data packets destined for the sink node(s). The average data packet generation rate is 0.2 packets per second or one packet per 5 s. Additionally, there are some constants that used by the WDFAD-DBR algorithm, e.g., weighting factor α and global parameter τ. The value of α=[0,1] prioritizes the depth differences (between *Sender* and *receiver* node and the receiver itself and its lowest depth neighbor) in the holding time computation of the WDFAD-DBR. To equality prioritize both the depth differences, we used α=0.5. Similarly, the global parameter τ had been used to guarantee the holding time between two nodes should be large enough to guarantee packet suppression. About 100 simulations of a single network scenario (a network with the specific number of nodes and the transmission range) are performed. The final results are the average of simulation trials and presented with the 95% confidence interval in the graphs. Simulation duration for a single sound was 500 s. The performance metrics that are measured through simulations are discussed in detail in the next section.

### 4.1. Broadcasted Copies of Data Packet:

The first performance metric that we investigate here is the number of copies forwarded in the network for all data packet transmissions initiated by the source node.

**Figure 5:**
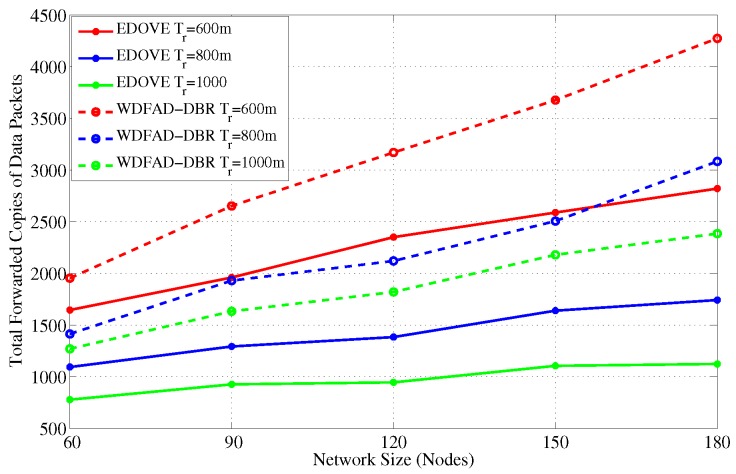
Number of forwarded copies of data messages in the network for different network sizes.

**Figure 6:**
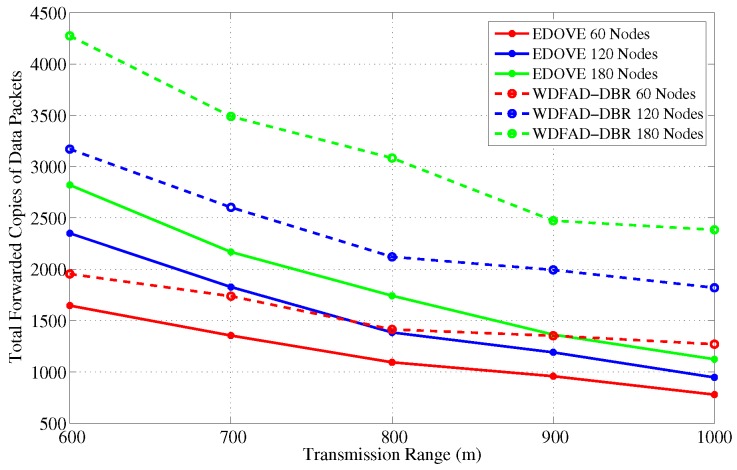
Number of forwarded copies of data messages in the network for different transmission ranges.

Figure 5 and Figure 6 show the total forwarded copies of data packets in the network for different size networks and transmission ranges, respectively. It is observed from Figure 5 that the number of forwarded copies of data packets in the network is directly proportional to the network size for the fixed transmission range. This phenomenon is obvious because in the large network size there may be many duplicate data packet transmissions. It is because the small network size and fixed transmission range do not cover the whole network, therefore, when the network size increases, more copies of the data packets are forwarded within the entire network area. In addition to that, there is a high probability of different nodes with similar depth difference and the depth difference between their next hop neighbors when network size increases. Hence, their estimated holding times are almost similar that increases the data packet traffic. In an extreme case, packet suppression can be guaranteed by both WDFAD-DBR and EDOVE, where there are multiple nodes at similar depths but extreme ends of node *S*’s transmission range $t_{r}^{S}$. These nodes are not in each other’s transmission range and can not suppress the duplicate packet forwarding. This is another reason that most of the nodes in the network just forward the packet.

**Figure 7:**
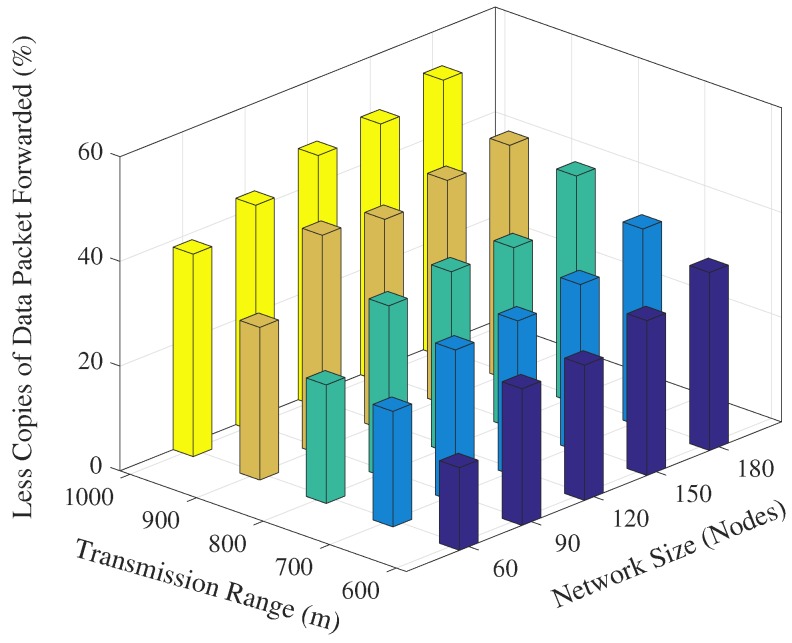
Data packet forwarding load reduced by EDOVE compared to WDFAD-DBR.

The next interesting trend that has been observed is that the number of forwarded copies of the data packets are inversely proportional to the transmission range in the fixed network size scenario. This phenomenon is also apparent due to the fact that the packet suppression is achieved at many nodes in the network when transmission range increases, refer Figure 6. However, it is not possible to guarantee the perfect packet suppression where only one node within the $t_{r}^{S}$ forwards the data packet. In result, the chances of packet collision may increase when the transmission range increases because of the large node density within the transmission range. Upon closely observing the simulation results, it is evident that the proposed EDOVE forwards less copies of data packet compared to WDFAD-DBR in any variation of the network parameter scenario. The WDFAD-DBR assigns constant priorities to the depth differences, which does not guarantee the enough holding time difference between the similar depth difference nodes that are the neighbors of the data forwarder. Hence, most of the neighbors of the data forwarder further relay the data packet. This also increases the packet collision chance and reduces the packet delivery ratio, which is discussed later in this section. Figure 7 shows the performance of EDOVE in terms of how much less copies of data are propagated within the network compared to WDFAD-DBR. It shows that EDOVE propagates about 53% less copies of the data packet than WDFAD-DBR for the largest network size and transmission range. From the above analysis, it is clear that the EDOVE reduces data broadcast storm for varying network sizes and transmission ranges. Next, we analyze the packet delivery performance of the EDOVE and WDFAD-DBR.

### 4.2. Packet Delivery Ratio-PDR

It is the ratio of successfully received data packets by the sinks and the total number of generated data packets by the source node. Let, $K_{1}$ denotes the set of successfully received data packets received by sink 1 and there are *m* sinks and *n* data packets generated, the PDR is denoted as:

$$PDR=\frac{\left|\overset{m}{\underset{j=1}{⋃}}K_{j}\right|}{n}$$

**Figure 8:**
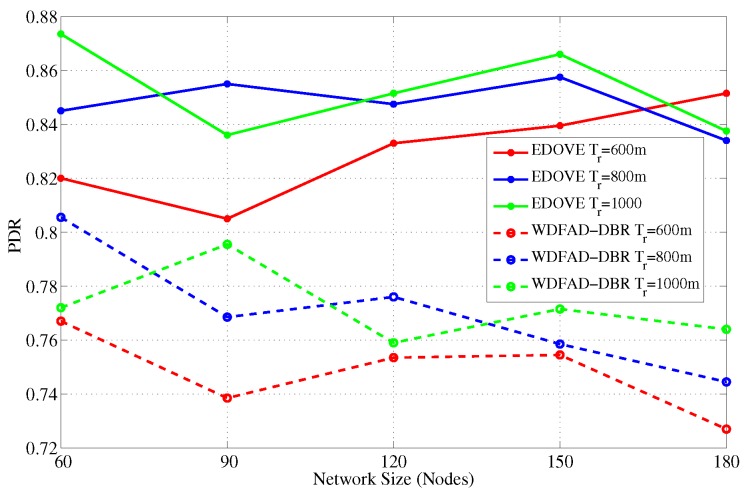
Average packet delivery ration versus the network sizes.

**Figure 9:**
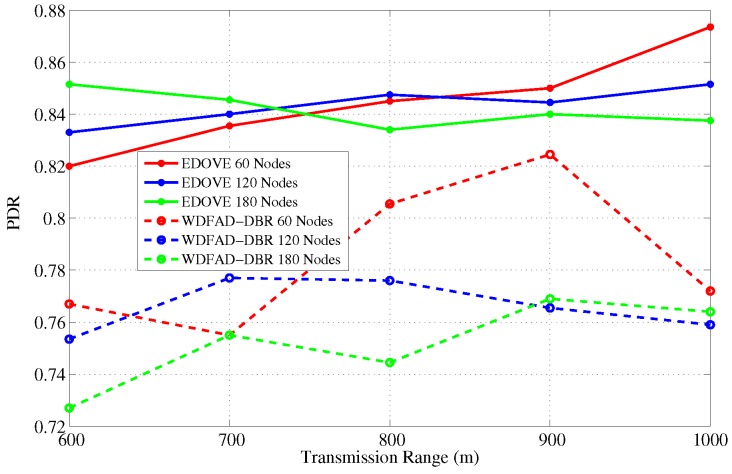
Average packet delivery ration versus the transmission range.

The main objective of any routing or forwarding scheme is to improve different performance metrics of the network and PDR is one of them. Therefore, we also analyzed and discussed the PDR performance metric of the EDOVE scheme. PDR performance of the EDOVE and WDFAD-DBR for varying network sizes and transmission ranges are shown in Figure 8 and Figure 9, respectively. Figure 8 shows that the PDR of the EDOVE is improved compared to the WDFAD-DBR for different size networks. As we discussed the phenomenon previously that the number of network-wide forwarded copies of data packets is directly proportional to the network size for the fixed $t_{r}$. Because of the large data packet transmissions, the packet collision probability becomes high, and the energy of the intermediate relaying nodes depletes quickly. As most of intermediate forwarder nodes’ battery power depletes, they could not forward the packets. As EDOVE fairly selects forwarders by using their normalized residual energy in the holding time computation, it avoids penalizing the low battery power nodes in the network. Hence, the network using EDOVE survives longer than the network that uses the WDFAD-DBR scheme. The energy consumption and the number of dead nodes are also analyzed and discussed later in this section. The dead nodes create a void region and/or a layer is created that completely disconnects the source node from the sink(s). These events collectively result in a drastic reduction in the PDR. Additionally, it can also be observed from the figure that as the network size increases, PDR of EDOVE either remains constant or slightly increases, which is not the case of WDFAD-DBR.

Next, in the fixed network size and variable transmission range scenario, as the transmission range increases node density increases as well, refer Figure 9. This increases the probability of successfully forwarding the data packets toward the sink(s) because a large number of redundant transmissions can be suppressed as well as the number of hops between source and sink(s) decreases. This increase in suppression and decrease in hop distance, increases the network lifetime, does not immediately create any void region, and more packets are successfully forwarded towards the sink. On average, EDOVE has 17% more PDR than WDFAD-DBR for any size network and $t_{r}=600$ m. Similarly, EDOVE improves about an average of 12% PDR for any $t_{r}\geq 700$ m contrast to WDFAD-DBR.

### 4.3. Energy Consumption and Energy Tax

**Figure 10:**
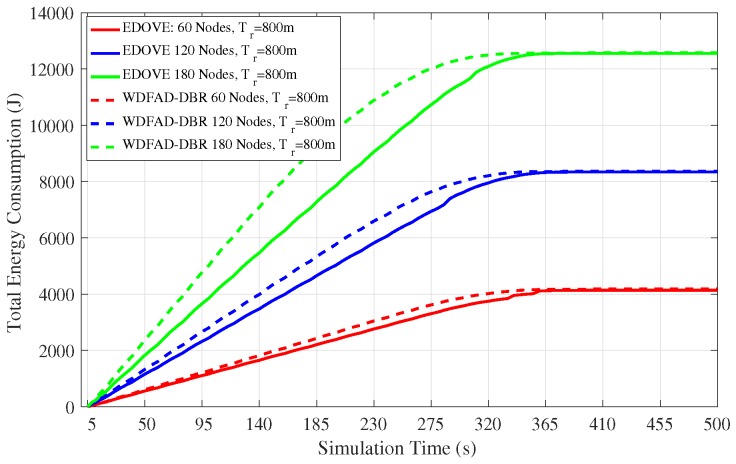
Total network energy consumption during the simulation duration for fixed transmission range of 800 m and varying number of nodes in the network.

In addition to data broadcast overhead and PDR, we also analyzed the total energy consumption of the network as the simulation time progresses. The total energy consumption consists of transmission, reception and idle energy consumption of the network. This total consumed energy value is used in the normalized energy consumption that we discussed previously. This analysis shows the real behavior of the routing scheme proposed for the network. Figure 10 depicts the total network energy consumption for the fixed transmission range and different network sizes for the complete simulation course. It is evident from the figure that the total energy consumption of the network is directly proportional to the network size. However, the proposed EDOVE consumes less energy than the WDFAD-DBR.

**Figure 11:**
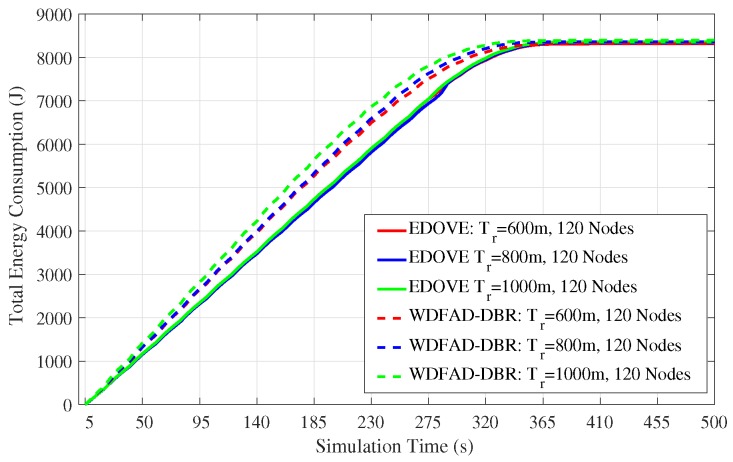
Total network energy consumption during simulation duration for the fixed number of nodes (120 nodes) and different transmission ranges in the network.

Next, the total energy consumption of the network for the fixed network size and different transmission ranges throughout the simulation course is shown in Figure 11. It shows that there is not that much impact of variation of the $t_{r}$ on the energy consumption of the EDOVE. However, it is noticeable from the same graph that WDFAD-DBR consumes slightly less energy for the $t_{r}=600$ m than the $t_{r}=1000$ m. In addition to that, it is also apparent from the figure that after 350 s the energy consumption become flat, which shows that there is no more data forwarding in the network. The main reason behind this occurrence is due to the fact that there has been a void layer or most of the relaying nodes have been depleted and no more data could be forwarded towards the sink(s). Energy consumption of the WDFAD-DBR saturates earlier than the EDOVE, which shows that most of the relaying nodes in WDFAD-DBR run out of the battery power earlier. In the context of this paper, we call this theory as the operation time that is discussed later in this paper. The operational time is the duration through which data packet can be forwarded from the source node towards the sink(s).

In addition to the network energy consumption analysis, we also compared the proposed EDOVE with WDFAD-DBR in terms of the *energy tax*, $E_{Tax}$. WDFAD-DBR computes energy tax as the average energy consumed per successfully received data packet by the sink(s) and per network node.

(7) $$E_{Tax}=\frac{E_{Total}}{P_{Success}\times Nodes}$$

where, $P_{Success}$ is the total number of successfully received packets at sink node(s). In case of multiple sinks, $P_{Success}$ is the distinct data packets that are received by multiple nodes. It is believed the WDFAD-DBR authors that energy tax parameter is more meaningful than total energy consumption. Hence, we compared the performance of EDOVE with the WDFAD-DBR in terms of energy tax for varying network sizes and transmission ranges, which is discussed below.

**Figure 12:**
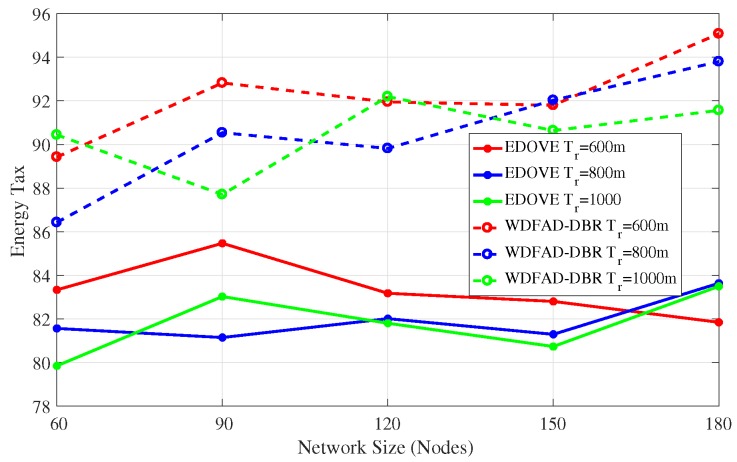
Energy Tax of the network versus the network sizes.

**Figure 13:**
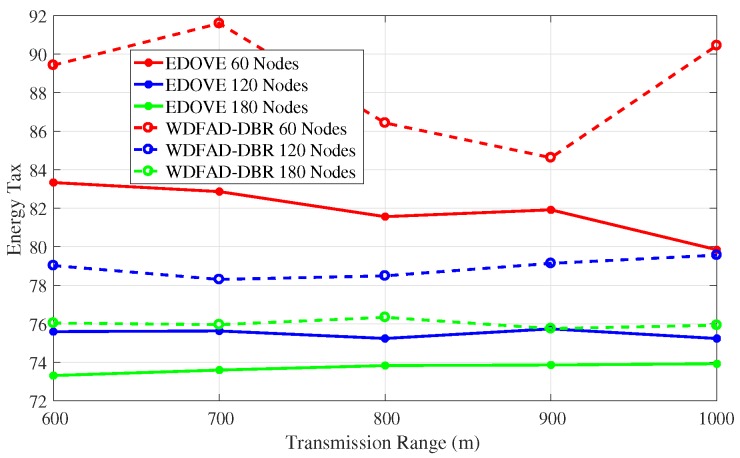
Energy Tax of the network versus the transmission range.

The energy tax analysis of the EDOVE for different network sizes and transmission ranges are shown Figure 12 and Figure 13, respectively. It shows that the energy tax of the EDOVE is far less than the WDFAD-DBR for different network sizes. The main reason for this behavior is that WDFAD-DBR consumes more energy and has very less delivery ratio. In result, it has very high energy tax compared to the EDOVE scheme. However, it is worth noting that the energy tax trend for both the schemes slightly increases as the network size increases. It is only because the volume of energy consumed due to large copies of data are disseminated is very high and number of generated data packets by the source node are same for any network size. The simulations also proved that the transmission and reception of these data packets have large impact on the overall energy consumption of the network.

### 4.4. Number of Dead Nodes

**Figure 14:**
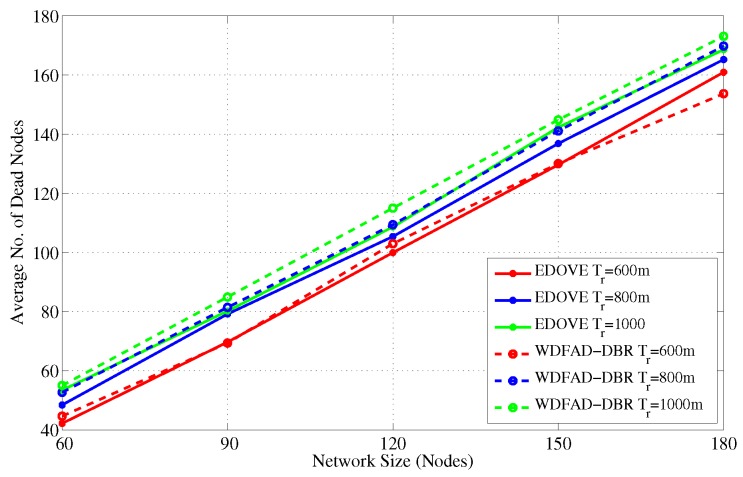
Number of dead nodes versus different network sizes.

A node is considered dead when its battery is depleted completely and it could not participate in the data forwarding process. Figure 14 shows the average number of dead nodes for different network sizes and fixed transmission ranges. It indicates that the number of dead nodes increases as the network size increases and that is obvious because of a large number of nodes participating in the forwarding process. In all the network scenarios with different network sizes, the number of dead nodes in the EDOVE is less than the WDFAD-DBR. However, at some cases e.g., network of 180 nodes, the number of dead nodes in EDOVE scheme are larger than the conventional WDFAD-DBR. This is because of the EDOVE’s fair distribution of data forwarding load on most of the nodes that enable these nodes to be active for a longer time and resulted in the large PDR, refer discussion related to PDR in the previous subsection.

**Figure 15:**
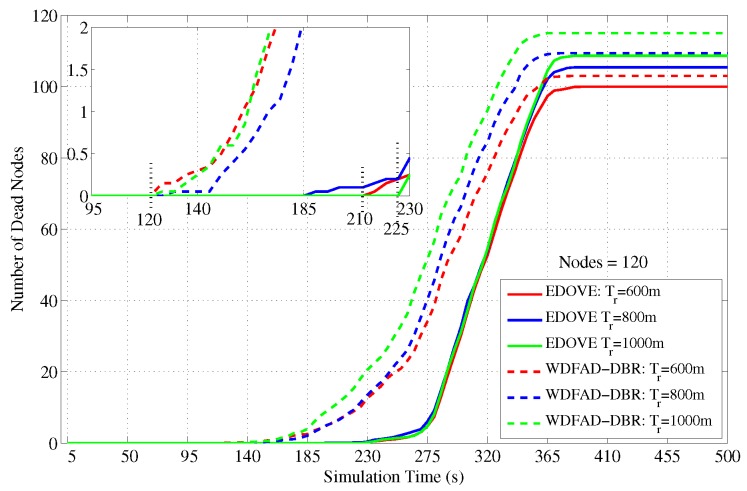
Number of dead nodes during simulation duration for the fixed transmission range and different network sizes.

The next detailed analysis related to the dead nodes is shown in Figure 15 that shows the total number of dead nodes over the course of the simulation. In the case of WDFAD-DBR, the nodes start dying out immediately after 120 s of the simulation time. On the other hand, the nodes start dying or depleting their batteries between 180 s to 230 s when EDOVE scheme is executed on the same network scenario because it fairly distributes the data forwarding load using their residual energy and the distribution of their second hop potential forwarders. It shows that the network using EDOVE survives for a longer duration than the WDFAD-DBR and forwards more data packets towards the sink(s).

**Figure 16:**
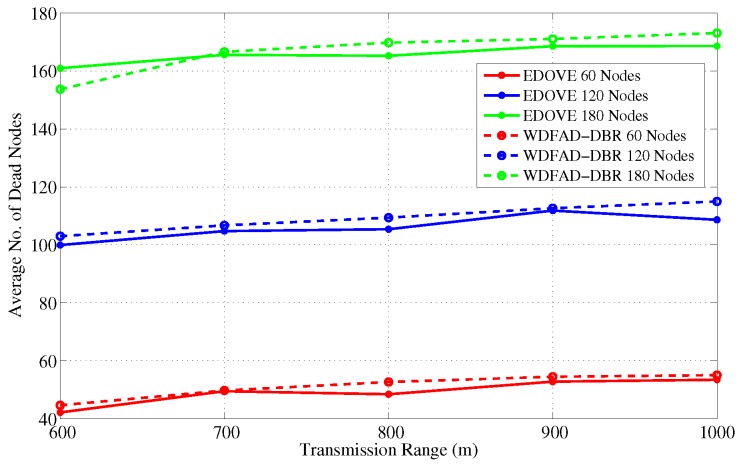
Number of dead nodes versus different transmission ranges.

**Figure 17:**
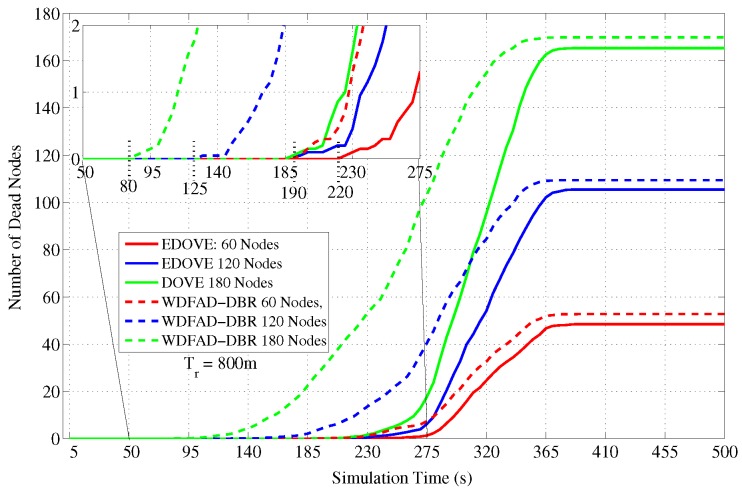
Number of dead nodes during simulation duration for the fixed network size and different transmission ranges.

Furthermore, we simulated the average number of dead nodes for the varying transmission ranges and different network sizes with a fixed transmission range of 800 m over the course of simulation, as shown in Figure 16 and Figure 17, respectively. Figure 16 shows that the average number of the dead nodes increases as the network size increases, which is only because the data packet traffic drastically increases with the increase in the network size, refer Figure 5. However, it can easily be observed from the figure that EDOVE has slightly less number of dead nodes than the WDFAD-DBR. The number of dead nodes is an important factor. However, the main factor is the time instance when these nodes die out during simulation. If they die out earlier during the simulation, then they can not properly participate in the data packet forwarding process. The number of dead nodes are analyzed for different network sizes and fixed $t_{r}=800$ m during the simulation course are shown in Figure 17. Due to high data packet traffic for the large network size, it can easily be seen from the figure that nodes start dying earlier during the simulation. For the network of 180, 120 and 60 nodes, the nodes start dying at 80 s, 125 s and 190 s, respectively, during the simulation course for WDFAD-DBR. On the other hand in the case of EDOVE, the nodes start to die out at the time instance of 185 s, 190 s and 220 s, for the network of size 180, 120 and 60, respectively. From the results, we can easily infer that the network using the EDOVE have slowed or delayed battery depletion rate.

### 4.5. Average Operational Time

**Figure 18:**
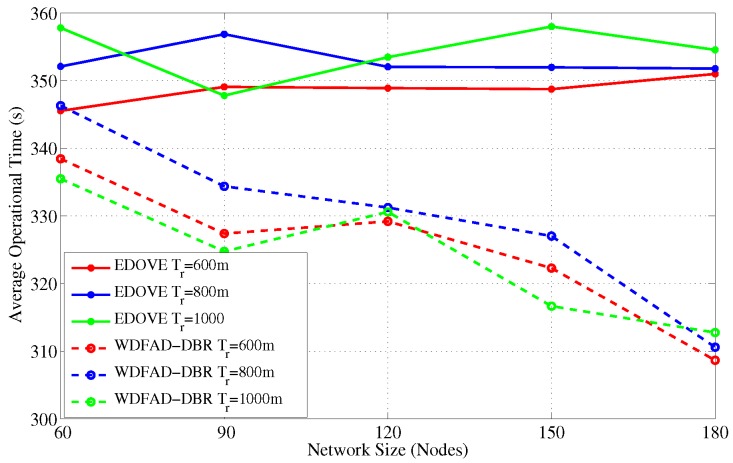
Average network operational time versus the network sizes.

**Figure 19:**
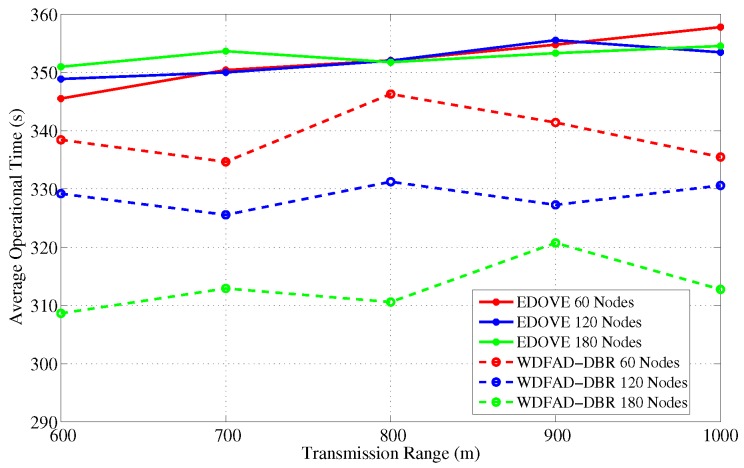
Average network operational time versus the transmission range.

The operational time is the time instance during the simulation when any of the sink(s) received the *last* data message from the source node. Figure 18 and Figure 19 show the operation time comparison of the EDOVE and WDFAD-DBR for varying network sizes and transmission ranges, respectively. From the previous discussion, it is clear that the nodes in WDFAD-DBR deplete their energy quickly because it does not consider any energy information in the forwarding decision. Hence, it fails to maintain energy fairness and potentially creates or extends the void area or void layer. In this case, no further communication can be carried out towards the sink and the time instance when the very last data message is received by the Sink is recorded and compared. Figure 18 shows that the average operational time of EDOVE is larger than the WDFAD-DBR. It is also evident from the figure that EDOVE has almost constant operational time for any size network. On the contrary, WDFAD-DBR’s operational time decreases with the increase in the network size. It is only because the WDFAD-DBR generates more data traffic and depletes energy of most of the nodes. Hence, most of the nodes near or between the source and sink node deplete their energy and fail to forward more data toward the sink.

**Figure 20:**
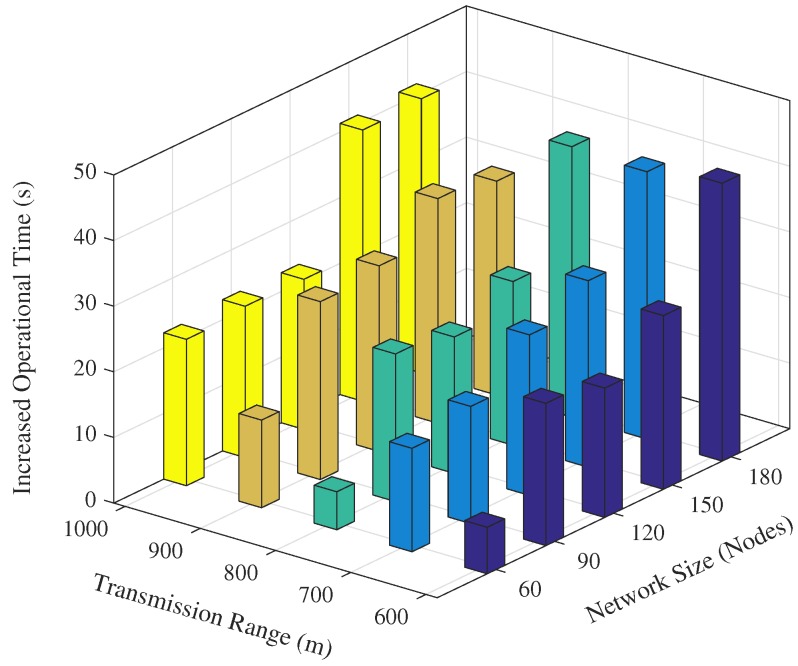
Overall increase network lifetime by the proposed EDOVE.

Similarly, we recorded the average operational time for the varying transmission ranges and show them in Figure 19. It is evident from the figure that operational time of the proposed scheme slightly increases as the transmission range increases. However, no marginal variation in the operational time for different network sizes was observed for the EDOVE. On the other hand, a significant change in the operational time of WDFAD-DBR was observed. It demonstrated the large operational time for the smaller The reason of the large operation time for smaller network size is that it generates less data packet broadcast. However, the simulation results demonstrate that overall operational time of the network using EDOVE is larger than by using the WDFAD-DBR protocol. The operational time of the EDVOE is almost 40 s more than of the WDFAD-DBR. The overall improvement of the proposed EDOVE in contrast to the WEDFAD-DBR is shown in Figure 20.

**Figure 21:**
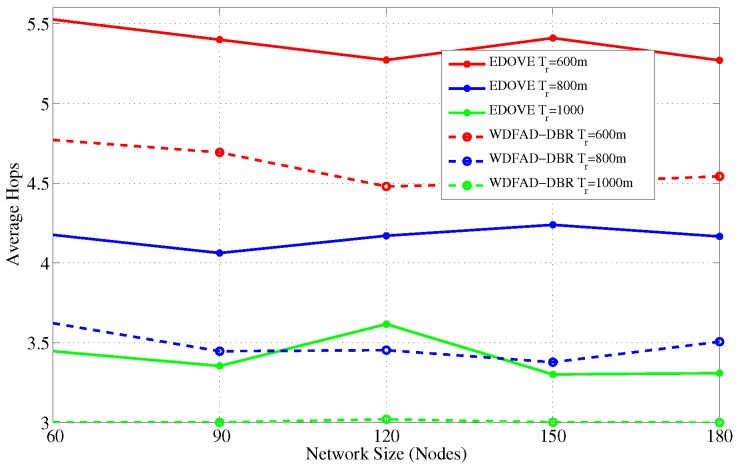
Average No. of Hops data messages traversed versus the network sizes.

**Figure 22:**
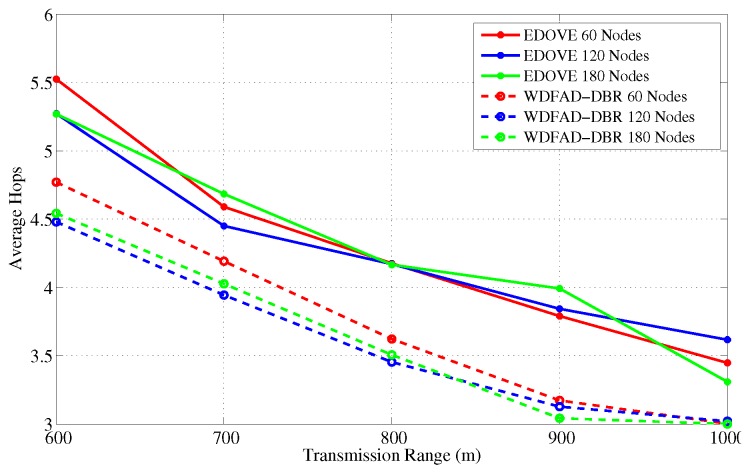
Average No. of Hops data messages traversed versus the transmission range.

**Figure 23:**
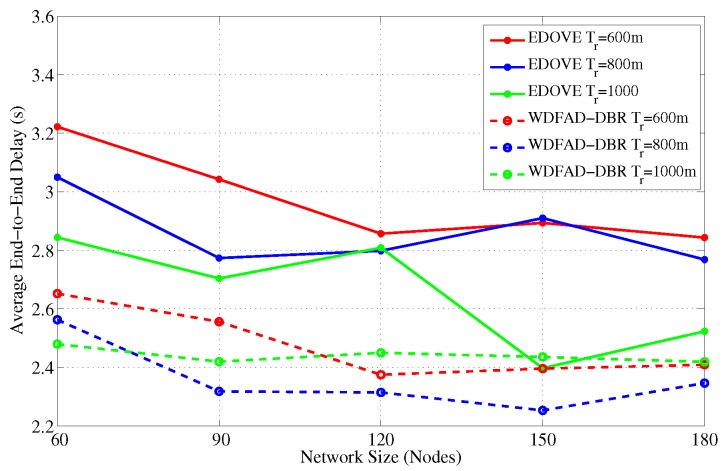
End-to-End delay between Source node that generated data message and Sink node versus the network sizes.

**Figure 24:**
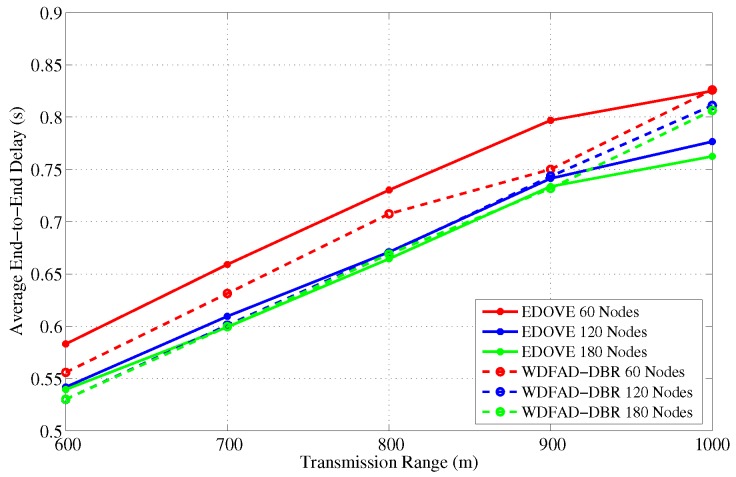
End-to-End delay between Source node that generated data message and Sink node versus the transmission range.

It has been evident from all the previous results and discussion that EDOVE has high PDR, less data broadcast, consumes less energy, fewer number of dead nodes, and has large operational time. Next, we investigate the average number of hops the data packet is forwarded from the source to sink node(s). Figure 21 and Figure 22 show the average number of hops for different network sizes and transmission ranges, respectively. These figures show that EDOVE has a slightly larger number of hops than the WDFAD-DBR. The reason for this slightly larger number of hops in EDOVE is that it focuses on the reduction of network’s energy consumption and increase network lifetime. This is achieved by EDOVE because it uses node’s residual energy, the variance of the neighboring nodes’ depths and depth differences between the sender, receiver, and receiver’s next hop forwarders. On the contrary, the WDFAD-DBR just considers the depth difference to select the next data packet forwarder, which reduces the number of hops. This is the reason that WDFAD-DBR has less End-to-End delay compared to EDOVE, refer Figure 23 and Figure 24 for different network size and transmission range.

Comprehensively, the proposed EDOVE scheme improves the PDR, reduces the data packet broadcast, conserves more energy which in turn reduces the number of dead nodes, and has more operational time than the WDFAD-DBR. However, it has slightly longer end-to-end hop distance and slightly larger delay, which is the tradeoff of the proposed scheme or may be any scheme proposed for the UASNs.

## 5. Conclusions

An energy and depth variance based opportunistic void avoidance (EDOVE) scheme for underwater acoustic sensor networks has been proposed in this paper. EDOVE uses the depth difference information between data packet sender to its one-hop neighbor and one-hop neighbor to its forwarders (that are two-hop neighbors of the *Sender* node). In addition to that, it prioritizes the depth differences using the normalized residual energy of the forwarder and the normalized depth variance of the two-hop forwarders to compute the holding time. In this manner, it offers energy fairness, distributes data forwarding load over the fairly distributed density nodes and increases the network lifetime. Simulation results show that EDOVE propagates about 53% less copies of the data packets, increases more than 40 (s) of network life time, reduces energy depletion of the nodes than the state of the art WDFAD-DBR. On the other hand, EDOVE has slightly longer delay because it focuses on the energy fairness and load balancing rather than the greedy depth based forwarding. This makes EDOVE to be a more suitable technique for UASNs.
